# Film, function, flexibility: label-free nanobody sensors *via* electropolymerized nanointerfaces

**DOI:** 10.1039/d6nr00562d

**Published:** 2026-04-10

**Authors:** Daniel P. Carroll, Imen Boumar, Anna M. Kotowska, David J. Scurr, Paula M. Mendes

**Affiliations:** a School of Chemical Engineering, University of Birmingham, Edgbaston Birmingham B15 2TT UK p.m.mendes@bham.ac.uk; b School of Pharmacy, University of Nottingham Nottingham NG7 2RD UK

## Abstract

Nanobody-based biosensors promise exceptional molecular recognition and robustness, but their implementation has been limited by unstable and non-scalable surface chemistries. Here we introduce a materials platform that integrates stable electropolymerized tyramine nanofilms with label-free, non-faradaic electrochemical impedance spectroscopy for direct molecular detection. The electropolymerized nanofilms form amine-functionalized coatings that are electrically insulating yet chemically active, supporting site-specific covalent immobilization of nanobodies in controlled orientations. Target binding induces more than a 50% increase in the capacitance signal response, providing a distinct label-free signature of molecular recognition. The platform can be adapted to diverse bioreceptors and antifouling layers, offering a general route to chemically robust and scalable bioelectronic interfaces. By decoupling film conductivity from functional stability, this work establishes a new class of interfaces that bridge molecular design with label-free signal transduction for next-generation biosensing technologies.

## Abstract 2

Electropolymerized polytyramine nanofilms enable stable, label-free nanobody biosensing *via* non-faradaic impedance, delivering selective detection through controlled interfacial chemistry and capacitance-based readout.

## Graphical abstract

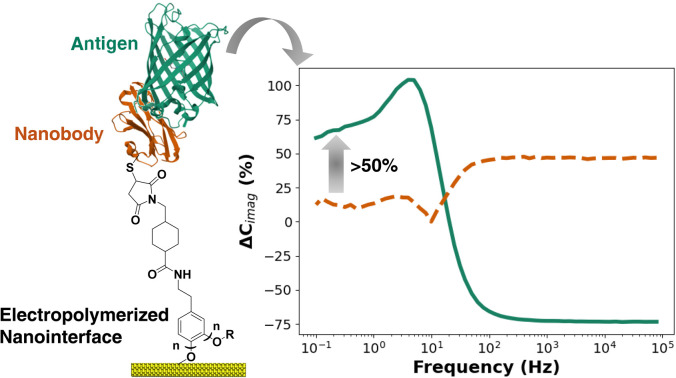

## Introduction

Nanobodies (Nbs) are single-domain antibody fragments derived from camelids, such as llamas and alpacas, and have emerged as powerful biorecognition elements for biosensing applications.^1–3^ Their compact size (∼15 kDa), excellent aqueous solubility, and remarkable thermal and chemical stability allow them to function under conditions that denature conventional antibodies, enzymes, or aptamers.^4,5^ For instance, Nbs targeting alpha-fetoprotein have demonstrated functional retention of over 60% after exposure to 90 °C, while IgG antibodies lose more than 90% of their activity under the same conditions.^6^ In addition to this intrinsic robustness, Nbs exhibit high specificity, ease of recombinant expression, and compatibility with site-specific engineering, making them ideal recognition elements for biosensors in complex or low-resource settings.^7,8^

Recent developments have highlighted the analytical versatility of Nb-based sensors across a broad spectrum of targets and platforms. Nbs against SARS-CoV-2 and MERS-CoV spike proteins have enabled attomolar-level detection in saliva using organic electrochemical transistor (OECT) sensors.^9,10^ Nb-functionalized nanopores have further demonstrated picomolar detection of both viral and cancer biomarkers by translating target-induced binding events into stochastic modulations of ionic current.^11^ In a separate study, site-specifically oriented Nbs grafted to silicon-based ellipsometry sensors enabled interleukin-6 detection at femtogram levels in serum, revealing clear distinctions between healthy and cancer patient samples.^12^ Beyond human health, Nb-based biosensors have also shown promise in food-safety and environmental applications, including the detection of enteric viruses such as rotavirus in wastewater,^13^ pesticide residues in water,^14^ and mycotoxins in food products,^15^ demonstrating their adaptability to complex sample matrices and field-deployable formats. While these examples underscore the capabilities of Nb-based platforms, their broader application remains constrained by interface limitations. The practical implementation of Nb sensors is often hindered not by the bioreceptors themselves but by the instability and complexity of the underlying immobilization strategies. Many systems rely on self-assembled monolayers or direct immobilization formed through thiol–gold chemistry,^16,17^ which are prone to degradation under oxidative or real-world conditions.^18,19^ Furthermore, uncontrolled attachment can compromise binding orientation, diminishing antigen accessibility and reducing sensitivity. Other sensing formats rely on recombinant fusion tags or require labelling with enzymes or redox mediators, which add complexity, extend assay time and limit scalability.^20^

These challenges underscore the need for detection strategies that operate without labels or additional molecular components. Non-faradaic electrochemical impedance spectroscopy (EIS) offers a compelling route for label-free detection by monitoring interfacial capacitance changes that arise directly from biomolecular binding events, eliminating the need for additional reagents. However, the sensitivity and reliability of non-faradaic EIS are critically dependent on the stability, insulation, and functionalization capacity of the underlying sensing layer.^21^ Existing coatings rarely provide all three attributes simultaneously, and many are incompatible with straightforward aqueous phase bioconjugation.

In response to these limitations, we introduce an electropolymerized polytyramine (Ptyr) interface that enables chemically robust, electrically insulating, and functionally versatile biosensing. Ptyr films form conformal, amine-rich nanocoatings on gold electrodes *via* electrochemical deposition, producing a stable surface for downstream biofunctionalization. The films are activated in aqueous conditions with a heterobifunctional crosslinker (Sulfosuccinimidyl-4-(*N*-maleimidomethyl)cyclohexane-1-carboxylate – SMCC), allowing site-specific coupling of cysteine-tagged Nbs without the need for organic solvents or recombinant fusion strategies. Using anti-enhanced green fluorescent protein (eGFP) Nbs as a model, we demonstrate selective and reproducible detection of the target antigen, with binding events generating a substantial increase in the imaginary component of the capacitance response. To establish the structural and chemical integrity of the sensing interface, surface-sensitive characterization techniques were employed that directly probe parameters relevant to non-faradaic EIS and that preserve the native structure of nanometer-scale organic and biomolecular layers formed on bulk gold electrodes. Ellipsometry was used to assess polymer film thickness, time of flight secondary ion mass spectrometry (ToF-SIMS) to resolve molecular composition and lateral uniformity of the interface, and X-ray photoelectron spectroscopy (XPS) to monitor chemical evolution during sequential functionalization. By decoupling conductivity from surface stability, our platform establishes a new framework for designing reliable and adaptable label-free biosensors.

## Results and discussion

Non-faradaic EIS detects molecular binding through perturbations of the electrical double-layer (EDL) at the electrode–electrolyte interface, where even subtle changes in local permittivity or ion distribution produce measurable shifts in interfacial capacitance.^22^ The magnitude, stability, and interpretability of this signal depend critically on the physical and chemical uniformity of the electrode surface. Commercial electrodes, particularly screen-printed carbons and polycrystalline gold, typically exhibit nanoscale roughness, grain boundaries, and localised defects that distort the interfacial electric field.^23^ These heterogeneities create microdomains with different effective Debye lengths, generating capacitive “hot spots” and contributing to non-ideal frequency dispersion in impedance spectra. When modified with molecular layers, these defects can act as ion-permeable pathways, producing ‘leaky capacitance’ that obscures true binding-induced dielectric changes and undermines sensor reproducibility.

In a double-layer architecture comprising functionalization and biorecognition layers, the overall interfacial capacitance is dominated by the lowest-capacitance component.^24^ As a result, the polymer film must have high intrinsic capacitance with strong electrical insulation to preserve sensitivity in the non-faradaic regime. To meet these requirements, we employed electropolymerized Ptyr as the functionalisation layer. Unlike conjugated polymers such as polypyrrole or aryl diazonium films,^25,26^ Ptyr is intrinsically non-conductive, forming a dense, defect-resistant barrier that suppresses parasitic leakage currents while providing abundant amine groups for oriented nanobody coupling. This combination of uniform surface passivation and chemical accessibility is essential for achieving reproducible, high-fidelity non-faradaic detection.

Ptyr films were prepared by electrochemical polymerization of tyramine on gold electrodes using cyclic voltammetry in alkaline methanolic solution. Optimization of the Ptyr layer was essential for achieving a reproducible and defect-resistant dielectric film. We systematically varied the tyramine concentration and CV scan rates (Fig. S1), parameters known to strongly influence the thickness, crosslinking density and insulating quality of electrogenerated polymer films.^27^ Specifically, tyramine concentrations of 25 and 50 mmol L^−1^ were evaluated, CV scan rates were varied between 50 and 200 mV s^−1^, and polymerization was carried out using two CV cycles between 0 and 1.6 V. During the first anodic sweep, a broad oxidation wave emerged just below 1.0 V (Fig. S1A), corresponding to the oxidative coupling of tyramine and nucleation of the polymer film. The rapid attenuation of this peak in subsequent cycles indicates progressive passivation of the gold surface, as the growing insulating network increasingly restricts electron transfer. This self-limiting behaviour is characteristic of insulating electropolymerized coatings and is a strong indicator of dense, conformal film formation. Across the parameter space examined, a tyramine concentration of 25 mmol L^−1^ combined with two CV cycles between 0 and 1.6 V at 50 mV s^−1^ produced films with the most favourable balance of uniformity, insulating strength and inter-electrode reproducibility. Lower scan rates yielded more insulating Ptyr layers, while higher scan rates produced incomplete coverage (Fig. S1). Although the relative variability in charge transfer resistance (*R*_ct_) is greater under more insulating conditions, this reflects the increased sensitivity of dense Ptyr films to nanoscale differences in thickness rather than uncontrolled deposition and is accompanied by consistently higher passivation across independently prepared electrodes, as shown in Fig. S1B. The starting concentration of tyramine monomer (25 *vs.* 50 mmol L^−1^) had less of an impact on film density and reproducibility. The selected conditions therefore conserved reagents, minimized structural defects and ensured consistent dielectric behaviour across electrodes, an essential requirement for high-fidelity non-faradaic EIS, where even minor variations in film thickness or porosity can markedly influence interfacial capacitance and signal stability.

**Fig. 1:**
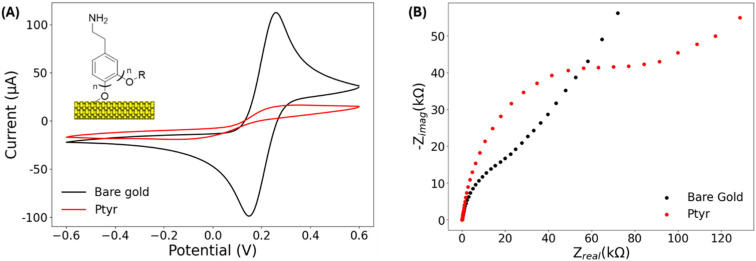
(A) CV and (B) EIS using 10 mmol L^−1^ Fe(CN)_6_^−3/−4^ in 1× PBS (0.1 mol L^−1^ KCl, pH 7.3–7.4) of the bare gold (black) and Ptyr (red) surface. Geometric electrode surface area = 0.03 cm^2^.

The effectiveness of the electropolymerized Ptyr film was first validated using cyclic voltammetry (CV) and faradaic EIS in the presence of 10 mmol L^−1^ ferri/ferrocyanide ([Fe(CN)_6_]^3−/4−^) in PBS. Relative to bare gold, Ptyr-modified electrodes displayed complete suppression of redox peaks (Fig. 1A), indicating that the polymer layer formed a continuous barrier preventing electron transfer to the redox probe. Correspondingly, *R*_ct_ increased from 8.2 ± 0.7 to 60.4 ± 17.1 kΩ (Fig. 1B), a hallmark of dense, pinhole-free dielectric films. The variability in *R*_ct_ between electrodes likely reflects minor differences in local film thickness inherent to electropolymerized coatings. However, all electrodes exhibited the same qualitative behaviour, confirming robust suppression of redox probe access.

Ellipsometry further supported the formation of a conformal and uniform polymer layer, revealing a film thickness of 5.4 ± 0.3 nm. This architecture is noteworthy: it is sufficiently thin to allow rapid ionic equilibration, critical for non-faradaic EIS, yet dense enough to eliminate leakage pathways that would otherwise introduce parasitic capacitance and obscure binding-induced dielectric changes. Together, these results establish a practical design rule for non-faradaic biosensors: maximising *R*_ct_ while maintaining nanometer-scale thickness yields the optimal balance between insulation and responsiveness. This level of electrical passivation provides a chemically stable, highly insulating interface ideally suited for subsequent Nb functionalization and sensitive capacitance-based detection.

**Fig. 2:**
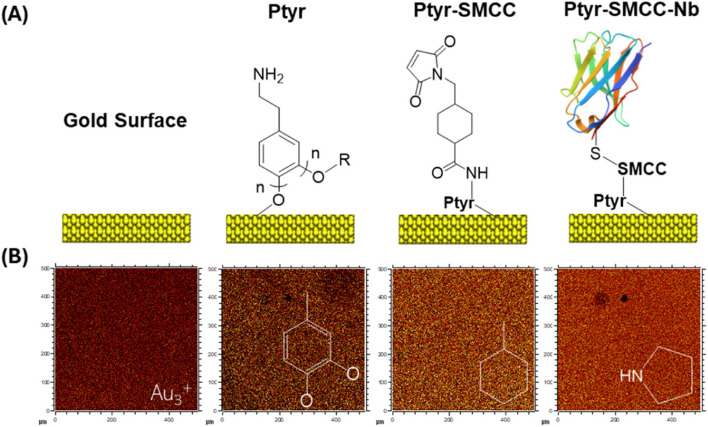
(A) Schematic representation of each surface modification stage: bare gold, electropolymerized Ptyr, SMCC crosslinker attachment (Ptyr–SMCC), and site-specific immobilization of cysteine-tagged Nbs (Ptyr–SMCC–Nb). (B) ToF-SIMS spatial distribution maps of characteristic fragment ions: Au_3_^+^ (gold), dihydroxybenzyl fragment C_7_H_5_O_2_^+^ (Ptyr), cyclohexyl C_7_H_11_^+^ (SMCC), and amino acid fragment C_4_H_8_N^+^ (Nb).

To enable covalent and site-specific attachment of Nbs (Fig. 2A), the amine-rich Ptyr surface was activated with the heterobifunctional crosslinker SMCC. This introduces terminal maleimide groups that selectively react with thiols under mild aqueous conditions, forming stable thioether linkages without perturbing the underlying polymer film.^28^ As the Nbs used here contain a single, strategically positioned C-terminal cysteine, their coupling to the activated surface proceeds in a fully oriented manner, ensuring that the antigen-binding domain remains exposed and accessible. This thiol–maleimide strategy provides greater control and surface definition than conventional amine-based immobilization, producing a chemically well-defined interface that supports reliable non-faradaic EIS measurements, where probe position relative to the electrical double-layer strongly influences signal generation.

ToF-SIMS is a surface sensitive chemical analysis, which has previously been used to assess the molecular identity and distribution of protein biochips^29^ and Nb immobilization.^30^ Herein, ToF-SIMS was used to resolve chemical fingerprints associated with each stage of surface modification (Fig. 2B). Unique fragment ions were detected for the underlying gold (Au_3_^+^), the Ptyr layer (dihydroxybenzyl fragments, C_7_H_5_O_2_^+^), the SMCC linker (cyclohexyl-based species, C_7_H_11_^+^), and the immobilized Nb (amino acid fragments, C_4_H_8_N^+^). These fragments were evenly distributed across the scanned area, confirming homogeneous coverage and these diagnostic ions were representative to their respective functionalisation steps (Fig. S2).

Principal component analysis (PCA) is a multivariate approach widely used to discriminate between ToF-SIMS spectra from different surface chemistries^31^ and was applied here to identify fragment ions characteristic of each functionalization step. As shown in Fig. S3, PCA clearly separated the spectra corresponding to each modification stage, highlighting the progressive chemical evolution of the interface.

To further demonstrate the stepwise construction of the sensing interface, XPS was performed at each surface modification stage to probe relative surface chemical states and their evolution during sequential functionalization. High-resolution spectra for bare gold, Ptyr, Ptyr–SMCC, and Ptyr–SMCC–Nb revealed progressive chemical changes across the Au 4f, C 1s and N 1s regions (Fig. S4). Relative elemental contributions and carbon bonding environments extracted from the integrated peak areas and deconvolution of the C 1s spectra are summarized in Table 1. Deposition of the Ptyr film led to a strong attenuation of the Au signal from 56.5% to 11.5%, accompanied by a sharp increase in the relative carbon contribution (from 43.5% to 81.9%) and the appearance of nitrogen at 6.6%. These changes confirm the formation of an amine-rich polymer layer that effectively shields the underlying gold. After activation with SMCC, the surface remained rich in carbon (83.9%), consistent with heterobifunctional linker incorporation. Subsequent immobilization of Nbs produced further enhancements in the relative C 1s and N 1s signals, indicative of the addition of a peptide- and amide-rich biomolecular layer. Deconvolution of the high-resolution C 1s spectra shows an increase in C–O/C–N and C=O components, accompanied by a corresponding decrease in the C–C contribution across each functionalization step. These trends reflect the progressive introduction of heteroatom-rich polymer, linker, and Nb layers. The relatively large variation observed for the C–C component on bare gold is attributed to adventitious carbon, which rapidly adsorbs upon exposure to ambient conditions prior to XPS analysis, resulting in spatially heterogeneous carbon coverage. In contrast, electropolymerization produces a homogeneous polytyramine film, leading to improved reproducibility.

**Table 1:** Relative surface elemental contributions and carbon bonding environments obtained from deconvolution of high-resolution XPS spectra for the bare gold surface, Ptyr, Ptyr–SMCC, and Ptyr–SMCC–Nb functionalized surfaces. Values are reported as mean ± standard deviation from four measurements acquired across two independently prepared samples

		Gold surface	Ptyr	Ptyr–SMCC	Ptyr–SMCC–Nb
Relative atomic percentages	Au	56.5 ± 2.3	11.5 ± 1.6	9.6 ± 1.7	8.3 ± 0.1
	C	43.5 ± 3.8	81.9 ± 1.7	83.9 ± 1.7	79.8 ± 3.3
	N	—	6.6 ± 2.0	6.5 ± 0.8	11.9 ± 1.7
Relative C percentages	C–C	81.4 ± 15.4	69.5 ± 5.9	71.4 ± 2.5	62.0 ± 10.9
	C–O/C–N	10.1 ± 5.0	17.9 ± 7.3	19.6 ± 1.0	22.7 ± 5.9
	C=O	8.5 ± 1.3	12.6 ± 5.9	9.0 ± 1.0	15.3 ± 1.0

Although the Nb includes a terminal cysteine, no sulphur signal was detected in the S 2p region, which can be attributed to the low sulphur content per unit area relative to the detection limits of XPS.^29^ Collectively, the consistent evolution of surface chemical states across the C 1s and N 1s regions provides compelling evidence for successful and sequential functionalization of the sensing surface.

**Fig. 3:**
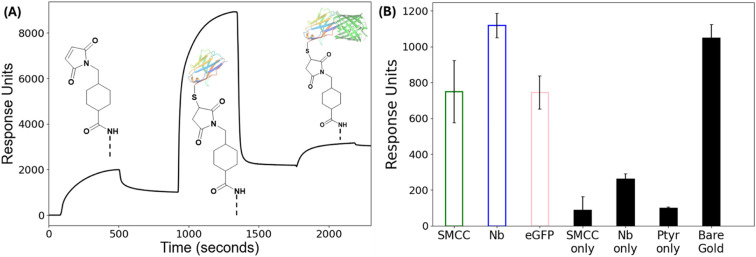
(A) Representative SPR sensorgram showing sequential injections of SMCC, Nb and eGFP onto a Ptyr-modified gold surface, recorded in 1× PBS at 25 °C. (B) Quantified changes in response units (RU) for each functionalization step (SMCC, Nb and eGFP) and after eGFP injection onto control surfaces, including: (i) SMCC activation without Nb coupling (SMCC only), (ii) Nb addition without prior SMCC activation (Nb only), (iii) unmodified Ptyr-coated surfaces (Ptyr only), and (iv) bare gold surfaces. All control surfaces were exposed to eGFP. Bars represent mean ± SD (*n* = 3).

To validate biomolecular activity and confirm successful Nb immobilization, surface plasmon resonance (SPR) was employed (Fig. 3). Ptyr-modified gold surfaces were functionalized with SMCC and sequentially exposed to the Nb and its eGFP target. Each step produced a clear increase in response units (RU), consistent with efficient layer-by-layer assembly and preserved binding functionality. Injection of SMCC resulted in a baseline shift of 750 ± 173 RU, indicating effective coupling to surface amines. Subsequent immobilization of Nbs yielded a further increase of 1119 ± 69 RU, confirming stable attachment through thiol–maleimide chemistry. Finally, exposure to the eGFP target produced an additional 745 ± 93 RU shift, demonstrating retained antigen-binding activity of the immobilized Nb layer. To assess specificity, eGFP was injected over control surfaces lacking individual functionalization steps, including (i) SMCC activation without Nb coupling, (ii) Nb addition without prior SMCC activation, (iii) unmodified Ptyr-coated surfaces, and (iv) bare gold electrodes. As shown in Fig. 3B, control surfaces lacking key functionalisation steps produced low RU shifts, with SMCC only, Nb only, and unmodified Ptyr surfaces yielding 153 ± 48 RU, 267 ± 58 RU, and 142 ± 35 RU, respectively. In contrast, the bare gold surface exhibited a substantially higher response of approximately 1100 RU, indicative of strong non-specific adsorption. These findings confirm that the interaction between the Nb and eGFP is specific, that both SMCC activation and thiol-reactive Nb coupling are required for effective target binding, and that the Ptyr layer serves as an effective antifouling coating by markedly reducing background signals. While the Ptyr layer reduces non-specific adsorption compared to bare gold under these conditions, more complex biological media such as serum or plasma present a more challenging fouling environment. The dense hydrophilic polymer layer provides a promising basis for antifouling, although dedicated studies in relevant biological matrices will be required to fully assess performance.

The nanobody–eGFP interaction employed here is based on a widely used GFP-binding nanobody with well-established high affinity. Representative biophysical studies using different experimental techniques report dissociation constants (*K*_D_) in the low nanomolar regime, with values on the order of ∼1 nM and method-dependent values spanning approximately 0.5 to 1.5 nM.^32^ These values provide a benchmark for the intrinsic affinity of GFP-binding nanobodies and confirm that the interaction strength is well within the range required for stable and specific target capture. Importantly, for surface-confined sensing architectures such as the one presented here, functional performance is governed not only by intrinsic affinity but also by geometric and interfacial factors including receptor orientation, surface density, and accessibility within the electrical double-layer. The small size of nanobodies (∼15 kDa), combined with site-specific and oriented immobilization *via* a single engineered C-terminal cysteine, minimizes steric hindrance and ensures consistent presentation of the antigen-binding interface. Such control over immobilization geometry is difficult to achieve with conventional antibodies, which are typically modified through random chemical labelling.^28^

**Fig. 4:**
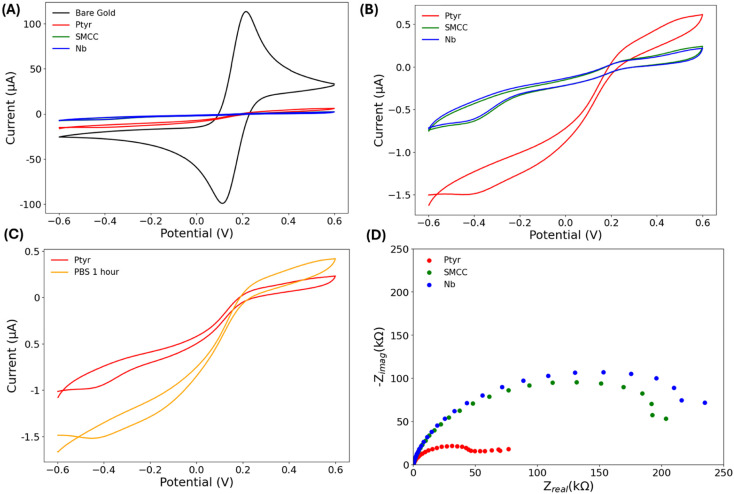
Electrochemical characterization of each surface modification step using 10 mmol L^−1^ [Fe(CN)_6_]^3−^/^4−^ in 1× PBS containing 0.1 mol L^−1^ KCl. (A) Cyclic voltammograms showing redox responses on bare gold and for each functionalization step (Ptyr, SMCC and Nb). At the scale shown, the SMCC (green) and Nb (blue) traces overlap closely and are not readily distinguishable. (B) Inset of (A) with the bare gold trace removed to better visualise subsequent modification steps. (C) Control cyclic voltammograms recorded after Ptyr deposition and incubation in 1× PBS without SMCC activation. (D) Nyquist plots from faradaic EIS measurements after each surface modification step. Geometric electrode area: 0.03 cm^2^.

To establish proof-of-concept in a practical sensing configuration, the full surface modification protocol was transferred to commercial gold rod electrodes. Each step was monitored electrochemically using CV and faradaic EIS in the presence of the [Fe(CN)_6_]^3−^/^4−^ redox couple. The bare gold electrode exhibited the characteristic, well-defined redox peaks associated with fast electron transfer (Fig. 4A). Upon deposition of the Ptyr film, these redox features were completely abolished, confirming that the polymer formed a continuous and highly insulating passivation layer. As expected for a dense dielectric coating, subsequent chemical modifications produced only subtle current changes, making them resolvable by CV only upon closer inspection. To better visualise the incremental changes in surface insulation, the bare-gold trace was removed from the overlay (Fig. 4B). Under these conditions, the SMCC activation step produced a clear further suppression of residual current, consistent with the addition of a second organic layer that increased interfacial resistance. Nb coupling caused no detectable change by CV, reflecting both the insulating character of the pre-existing film and the nanometer-scale thickness of the biomolecular layer. Control experiments performed in PBS without SMCC (Fig. 4C) confirmed that these changes originated from chemical modification of the surface rather than buffer-dependent artefacts.

Faradaic EIS provided more quantitative insight into the evolving interfacial structure. Upon SMCC activation, the *R*_ct_ increased markedly to ∼150 kΩ, consistent with the formation of a densely packed, non-conductive organic layer. Nb attachment produced a further, reproducible ∼25 kΩ increase, reflecting the additional steric and dielectric barrier imposed by the protein layer (Fig. 4D). These progressive increases in *R*_ct_ validate the stepwise build-up of the multilayer interface and demonstrate that the full fabrication protocol reliably produces well-defined, insulating surfaces on commercially available electrode formats.

eGFP detection was performed under non-faradaic conditions, where no redox probe is present and the sensing response is dominated by changes in interfacial capacitance. To ensure that binding-induced dielectric changes were captured within the electrostatically responsive region of the interface, all non-faradaic EIS measurements were conducted in low-ionic-strength buffer (0.001× PBS). Under these conditions, the Debye length is estimated to be approximately 23 nm based on bulk electrolyte properties, sufficient to encompass the full Nb–target complex, whose binding interface lies ∼10 nm from the electrode surface as inferred from ellipsometry and molecular dimensions. We note, however, that within the functionalized interfacial architecture, including the Ptyr and bioconjugation layers, the effective screening length may deviate slightly from this bulk value. In contrast, in 1× PBS the Debye length contracts to ∼0.7 nm, rendering protein–protein interactions largely electrostatically invisible. Modulating ionic strength therefore provides a straightforward means to position the biorecognition event within the capacitive sensing zone of the electrical double layer. Importantly, because molecular recognition itself occurs independently of the sensing environment, target capture can be performed in complex media, followed by a brief rinse into low-ionic-strength buffer for sensitive capacitive readout. To suppress unintended thiol–maleimide reactions during sensing, residual maleimide groups were quenched using 2-mercaptoethanol.

**Fig. 5:**
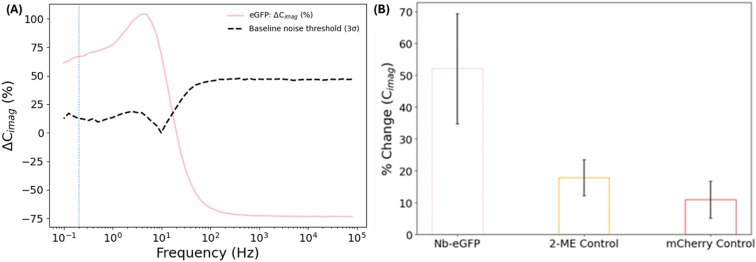
(A) Frequency-resolved differential capacitance response (Δ*C*_imag_ %) for Ptyr–SMCC–Nb functionalized electrodes recorded before and after incubation with 500 nmol L^−1^ eGFP. The dotted blue line indicates the selected analysis frequency (200 mHz), chosen based on frequency-resolved signal-to-noise and reproducibility considerations, from which the values in (B) were extracted. Baseline noise threshold (3σ), calculated from the standard deviation of the baseline signal prior to eGFP exposure. (B) Percentage change in *C*_imag_ at 200 mHz for Nb–eGFP compared with blocked control electrodes treated with 2-mercaptoethanol (2-ME) and Nb-functionalized electrodes exposed to the non-target protein mCherry. Data are shown as mean ± SD from independently prepared electrodes (*n* = 3). Electrode area = 0.03 cm^2^.

Under these optimised conditions, non-faradaic EIS revealed clear and reproducible changes in interfacial capacitance upon exposure to 500 nmol L^−1^ eGFP. Bode plots recorded before and after target incubation diverged markedly in the low-frequency regime (Fig. 5A), consistent with strong modulation of interfacial permittivity by biomolecular binding. Fig. 5A shows the full frequency-dependent response, from which the capacitance at 200 mHz was extracted for quantitative comparison. The imaginary component of impedance (*C*_imag_), which directly reports on interfacial capacitance, increased substantially following eGFP exposure, but only under low-ionic-strength conditions. In 1× PBS, no measurable change in *C*_imag_ was observed (Fig. S5), confirming that the Debye length must exceed the probe–target separation for capacitive transduction to occur.

*C*_imag_ emerged as a particularly informative and sensitive transduction parameter, enabling frequency-resolved analysis of the binding response. Although the differential response exhibits a maximum at intermediate frequencies, analysis at these frequencies is more susceptible to variability and fitting artefacts. Instead, 200 mHz (indicated by the dotted blue line in Fig. 5A) was selected as the operating frequency for quantitative analysis, as it provides a large, monotonic, and highly reproducible response while maximizing signal-to-noise across independently prepared electrodes. This approach allows sensitivity to be defined in terms of resolvable capacitance perturbations relative to interfacial variability, which is the most physically relevant metric for non-faradaic capacitive biosensing. At this frequency, Nb-functionalized electrodes exhibited a substantial mean increase of 52 ± 17% in *C*_imag_ following eGFP exposure (Fig. 5B).

To assess selectivity and exclude non-specific contributions, responses at 200 mHz were compared against two independent control conditions. Electrodes quenched with 2-mercaptoethanol and subsequently exposed to eGFP produced a significantly smaller response of 18 ± 6%, while Nb-functionalized electrodes exposed to the non-target protein mCherry exhibited a response of only 11 ± 6% (Fig. 5B). Despite the electrode-to-electrode variability reflected in the reported standard deviations, the separation between target and control responses substantially exceeds the associated measurement spread. This behaviour reflects the intrinsic sensitivity of non-faradaic EIS to nanoscale interfacial heterogeneity, which directly influences interfacial permittivity and capacitive signal generation. The observed variability is attributed to inter-electrode nanoscale heterogeneity in the electropolymerized Ptyr film, leading to small variations in film thickness, surface capacitance, and nanobody immobilization efficiency. The responses are normalized to the pre-binding baseline capacitance of each electrode and therefore represent relative changes in interfacial capacitance.

At the selected analysis frequency, the eGFP response is 2.9-fold greater than that observed for blocked control surfaces and 4.7-fold greater than that observed for the non-target protein, providing clear and reproducible discrimination under non-faradaic conditions. This high degree of selectivity highlights the combined advantages of an insulating, defect-free polymer interface, oriented Nb immobilization, and a buffer environment tuned to accommodate the spatial dimensions of the biorecognition complex. Taken together, these results demonstrate that electropolymerized Ptyr, when paired with non-faradaic EIS and Debye-length engineering, enables highly selective, label-free detection through subtle yet quantifiable shifts in interfacial capacitance. This strategy directly overcomes the classical Debye-screening limitation in electrochemical biosensing and establishes a generalisable framework for capacitive detection of protein targets at nanometer-scale distances from the electrode surface.

## Conclusion

This work introduces a stable and scalable materials platform that enables precise nanobody immobilization and direct interfacial capacitance readout of molecular binding. Electropolymerized polytyramine nanofilms provide an insulating yet chemically versatile interface that supports fully aqueous bioconjugation and removes the need for redox mediators or complex surface chemistries. Coupled with non-faradaic impedance spectroscopy, the platform delivers clear and selective signals arising solely from nanobody–antigen recognition. Its modularity, robustness and compatibility with diverse nanobody–target pairs position this approach as a strong foundation for next-generation, miniaturized biosensors capable of operating reliably outside controlled laboratory settings.

## Conflicts of interest

There are no conflicts to declare.

## Supplementary Material

NR-018-D6NR00562D-s001

## Data Availability

All data supporting this study are provided in the supplementary information (SI) accompanying this article. Supplementary information is available. See DOI: https://doi.org/10.1039/d6nr00562d.
