# Supplementary material for: Film, function, flexibility: label-free nanobody sensors via electropolymerized nanointerfaces
Source: Nanoscale. 2026 Apr 10;18(19):10326–34. doi: 10.1039/d6nr00562d (PMC13090008; doi:10.1039/d6nr00562d)
Supplement: NR-018-D6NR00562D-s001 [file NR-018-D6NR00562D-s001.pdf]

Supplementary Information (SI) for Nanoscale.
This journal is © The Royal Society of Chemistry 2026

SUPPORTING INFORMATION

Film, Function, Flexibility: Label-Free Nanobody Sensors via Electropolymerized Nanointerfaces

Daniel P. Carroll,[†] *Imen Boumar,*[†] *Anna M. Kotowska,*[‡] *David J. Scurr,*[‡] *Paula M. Mendes*[†,*]

[†]School of Chemical Engineering, University of Birmingham, Edgbaston, Birmingham, B15 2TT, UK.

[‡]School of Pharmacy, University of Nottingham, Nottingham, NG7 2RD, UK.

[*]Professor Paula M Mendes, School of Chemical Engineering, University of Birmingham, Edgbaston, Birmingham, B15 2TT, UK, Email: p.m.mendes@bham.ac.uk.

Materials and Methods

Chemicals

The following chemicals were purchased from Sigma Aldrich and used as received: Sulfuric acid (95-98%), hydrogen peroxide (30% aqueous solution), ethanol (HPLC grade, ≥ 99.9%), potassium hexacyanoferrate (III) (≥ 99%), potassium hexacyanoferrate (II) trihydrate (≥ 98.5%), potassium chloride (≥99%), tyramine (≥98%), 2-mercaptoethanol (≥99%), and 4-(N-maleimidomethyl)cyclo-hexane-1-carboxylic acid 3-sulfo-N-hydroxysuccinimide ester sodium salt. Enhanced-green-fluorescent protein (category number: EGFP) was purchased from

Chromotek. mCherry antibody (NBP2-25158) was purchased from Novus Biologicals. Nanobody specific for GFP with a C-terminal cysteine residue (Nb) were purchased from NanoTag Biotechnologies Ltd. (Germany). Phosphate buffered saline tablets were purchased from Thermo Fischer Scientific Ltd. One table was dissolved in 100 mL of water to create a 1x PBS solution. All references to water in this study refer to ultra-high purity water (resistance = approx. 17.6 MΩ.cm) obtained from a Milli-Q purification system.

Gold substrates

Gold sensor chips used in the SPR experiments were acquired from Reichert Technologies, Ametek Inc (USA) and consisted of polycrystalline gold surfaces (50 nm) on glass substrates sized 1 cm x 1 cm. For ellipsometry, X-ray photoelectron spectroscopy and ToF-SIMS studies, gold (100 nm thickness and rms roughness <2.5 nm) on silicon <100> wafers pre-coated with titanium were acquired from George Albert PVD (Germany) and cut into 1 cm x 1 cm pieces using a diamond scribe. For all electrochemical measurements, gold working electrodes (2 mm diameter, part number: CHI101P) were purchased from IJ Cambria Scientific Ltd. (UK).

Substrate treatment

The gold/silicon substrates were cleaned using piranha solution. Sulfuric acid and hydrogen peroxide were mixed in a shallow glass Petri dish in a 7:3 v/v ratio (10 mL total volume), surrounded by ice in a larger glass water bath. This mixture was allowed to react together for 10 min before the gold substrates were submerged in the piranha solution for 10 min. The substrates were then carefully removed using stainless-steel tweezers and washed with copious amounts of water and ethanol before being dried and stored under an argon atmosphere. The commercial gold rod electrodes were manually polished using alumina slurry (0.05 μm) on an electrode polishing pad for 1 min in a figure of 8 motion. They were then sonicated in ethanol for 10 min to remove

any excess alumina particles. Finally, they were cleaned electrochemically in 0.5 mol L^{-1} sulfuric acid by scanning the potential from -0.4 to 1.6 V until a stable cyclic voltammogram was obtained (usually around 12 cycles). The electrodes were then washed with copious amounts of water and ethanol and dried under a stream of argon.

Ellipsometry

Ellipsometry measurements were obtained with a J.A. Woollam alpha-SE instrument. Data analysis used the Cauchy model that considers three layers: Ambient/Monolayer/Substrate. The refractive index was fixed at 1.5. Each chip was measured before and after functionalization. Data was fitted with the software CompleteEASE, with a defined resolution of 0.1 eV. Four measurements per chip (duplicates) were performed in different chip locations.

Time-of-flight-secondary ion mass spectroscopy (ToF-SIMS)

ToF-SIMS spectra were acquired using a TOF 5 (IONTOF GmbH) instrument with 30 keV Bi_3^+ primary ion beam with a current of 0.06 pA. Two 256 × 256 pixel images over area of 500 × 500 µm were acquired on two replicates of each sample type. Measurements were performed in positive polarity due to the chemistry of the sample being composed of amino acids. Positive mode spectra were calibrated to: CH_3^+, $C_7H_7^+$, Au_3^+. Each measurement lasted 15 scans and the static limit was not exceeded during the analysis.

Surface Plasmon Resonance (SPR)

All SPR measurements were carried out using a Reichert Technologies SPR, operating at a wavelength of 780 nm. Before any experiments were carried out, the SPR equipment was flushed out with 0.5% sodium dodecyl sulfate (SDS), 50 mmol L^{-1} glycine pH 9.5 and water. The running buffer which was usually 1x PBS was prepared daily, which included filtering (0.2 µm filter) and

degassing of the solution. The temperature of the SPR was preset to 25°C for all measurements. The stage was cleaned with ethanol before each experiment and fresh immersion oil was then placed on the stage before the Au substrate to minimise the refractive index change. The SPR response was stabilized before each experiment using the running buffer with the initial flow rate set to 100 µL/min then decreasing to 30 µL/min after 10-15 min. Blank injections of the running buffer were then carried out before each experiment to remove any impurities and/or pockets of air in the injection loop. All SPR injections were pre-programmed and consisted of an initial 7 min loading phase which allowed the sample to be loaded into the injection loop. The injection phase also last 7 min with a flow rate of 30 µL/min using a 250 µL injection loop and finally, there was a dissociation or washing phase of the running buffer which lasted 7 min with a flow rate of 30 µL/min. SPR measurements were repeated on three independently prepared surfaces, and quantified responses are reported as mean ± standard deviation.

X-ray Photoelectron Spectroscopy (XPS)

XPS analysis was performed using a Thermo NEXSA XPS fitted with a monochromated Al kα X-ray source (1486.7 eV), a spherical sector analyser and 3 multi-channel resistive plate, 128 channel delay line detectors. All data was recorded at 19.2W and an X-ray beam size of 200 x 100 µm. Survey scans were recorded at a pass energy of 160 eV, and high-resolution scans recorded at a pass energy of 20 eV. Electronic charge neutralization was achieved using a dual-beam low-energy electron/ion source (Thermo Scientific FG-03). Ion gun current = 150 µA, ion gun voltage = 45 V. All sample data was recorded at a pressure below 10^{-8} Torr and a room temperature of 294 K. XPS peak fitting was performed using the CasaXPS v2.3.19PR1.0 processing software. Either a Shirley or linear background was used and the relative sensitivity factors for quantification were

built in to the CasaXPS library. Four measurements were performed, with two measurements acquired on each of two independently prepared samples.

Electrochemical Impedance Spectroscopy (EIS)

All electrochemical experiments were performed using a standard three-electrode configuration consisting of a gold working electrode, an Ag/AgCl (3 M NaCl) reference electrode, and a platinum wire counter electrode. This configuration was used for EIS, cyclic voltammetry and electropolymerization.

EIS measurements were performed at the open circuit potential (OCP) using a potentiostatic configuration. Faradaic EIS was carried out in 10 mmol L^{-1} $K_3Fe(CN)_6/K_4Fe(CN)_6$ in 1x PBS with 1 mol L^{-1} KCl. The input AC voltage was 10 mV, and the frequency was scanned from 100 KHz to 0.1 Hz at 10 points/decade. There was an initial voltage delay of 30 sec. An expansion on the Randles equivalent circuit was used to model the experimental data. Data analysis was carried out using Gamry Echem Analyst software. For non-Faradaic EIS the experimental parameter remained the same however, the redox probe was omitted from the electrolyte solution. Furthermore, no modelling of the non-Faradaic response was carried out, with single values reported at selected frequencies. All electrochemical measurements were performed on at least three independently prepared electrodes, and data are reported as mean ± standard deviation.

Cyclic Voltammetry (CV)

Cyclic Voltammetry was carried out using 10 mmol L^{-1} $K_3Fe(CN)_6/K_4Fe(CN)_6$ in 1x PBS with 1 mol L^{-1} KCl. The potential was scanned from 0.6 to -0.6 V at a scan rate of 100 mV/s for two cycles. The step size was 1 mV and there was an initial voltage delay of 30 sec. Data analysis was carried out using Gamry Echem Analyst software.

Tyramine Electropolymerizing

Tyramine (25 mmol L^{-1} or 50 mmol L^{-1}) was dissolved in 2 mL of methanol containing 300 mmol L^{-1} NaOH. Sonication was used to fully dissolve the tyramine. The solution was then poured directly into the electrochemical cell and the potential was scanned from 0 to 1.6 V for 2 scans with a scan rate of either 50, 100 or 200 mV/s. The working electrode was then rinsed with copious amounts of methanol and then phosphate buffered saline (PBS, pH 7.3-7.4).

SMCC Surface Activation

SMCC (5 mmol L^{-1}) in PBS (pH 7.3-7.4) was incubated on the tyramine derived film for 1 hour under gentle agitation. The surface was then washed with copious amounts of PBS (pH 7.3-7.4).

Nb Coupling

SMCC-activated electrodes were exposed to 3.6 μmol L^{-1} nanobody in PBS (pH 7.3–7.4) for 1 h. After rinsing with PBS, the surfaces were equilibrated in PBS overnight at 4 °C. To quench any unreacted maleimide groups, immobilized nanobody surfaces were subsequently treated with 1 mmol L^{-1} 2-mercaptoethanol (MEA) in deionized water for 30 min. Negative controls were prepared by incubating 1 mmol L^{-1} MEA in deionized water directly on SMCC-activated surfaces for 30 min. All surfaces were then washed with PBS and allowed to stabilize in PBS overnight at 4 °C.

eGFP Incubation

250 μL of eGFP stock solution (1 g/L) was received from ChromoTek and immediately separated into 20 μL aliquots and stored at -20˚C. Each 20 μL aliquot

of eGFP stock was then solubilized in 1408 μL 1x PBS (pH 7.3-7.4) to obtain a final eGFP concentration of 14 μg/mL or 500 nmol L^{-1}. The eGFP solution was incubated on the surface for 30 min with gentle agitation by pipetting every 5 min. The surface was then rinsed with copious amount of 0.001x PBS to remove any non-specifically bound eGFP. Negative controls were carried out using a mCherry antibody (1:100 dilution) in PBS, which was incubated for 30 min under identical agitation and washing conditions.

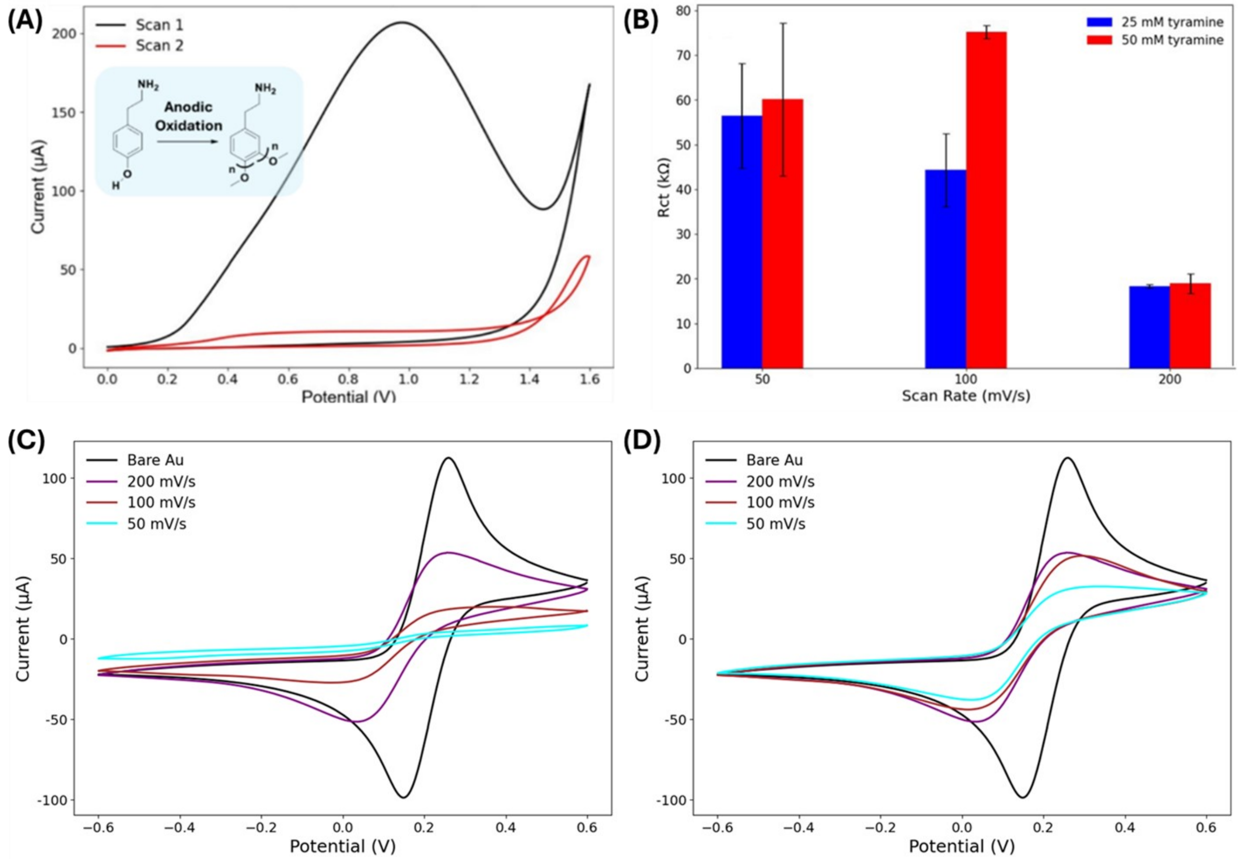

Figure S1 - (A) Cyclic voltammogram of 25 mmol L^{-1} tyramine in a 300 mmol L^{-1} NaOH methanol solution, scan rate = 50 mV/s. **(B)** Rct values of 25 and 50 mmol L^{-1} tyramine electropolymerized for 2 cycles between 0 and 1.6 V at 50, 100, and 200 mV/s. (mean ± SD, n = 3) Cyclic Voltammetry using 10 mmol L^{-1} $Fe(CN)_6^{-3/-4}$ in 1x PBS (0.1 mol L^{-1} KCl, pH 7.3-7.4) after polytyramine formation at 50, 100, and 200 mV/s with **(C)** 25 mmol L^{-1} and **(D)** 50 mmol L^{-1} tyramine monomer starting concentration. Geometric electrode surface = 0.03 cm^2.

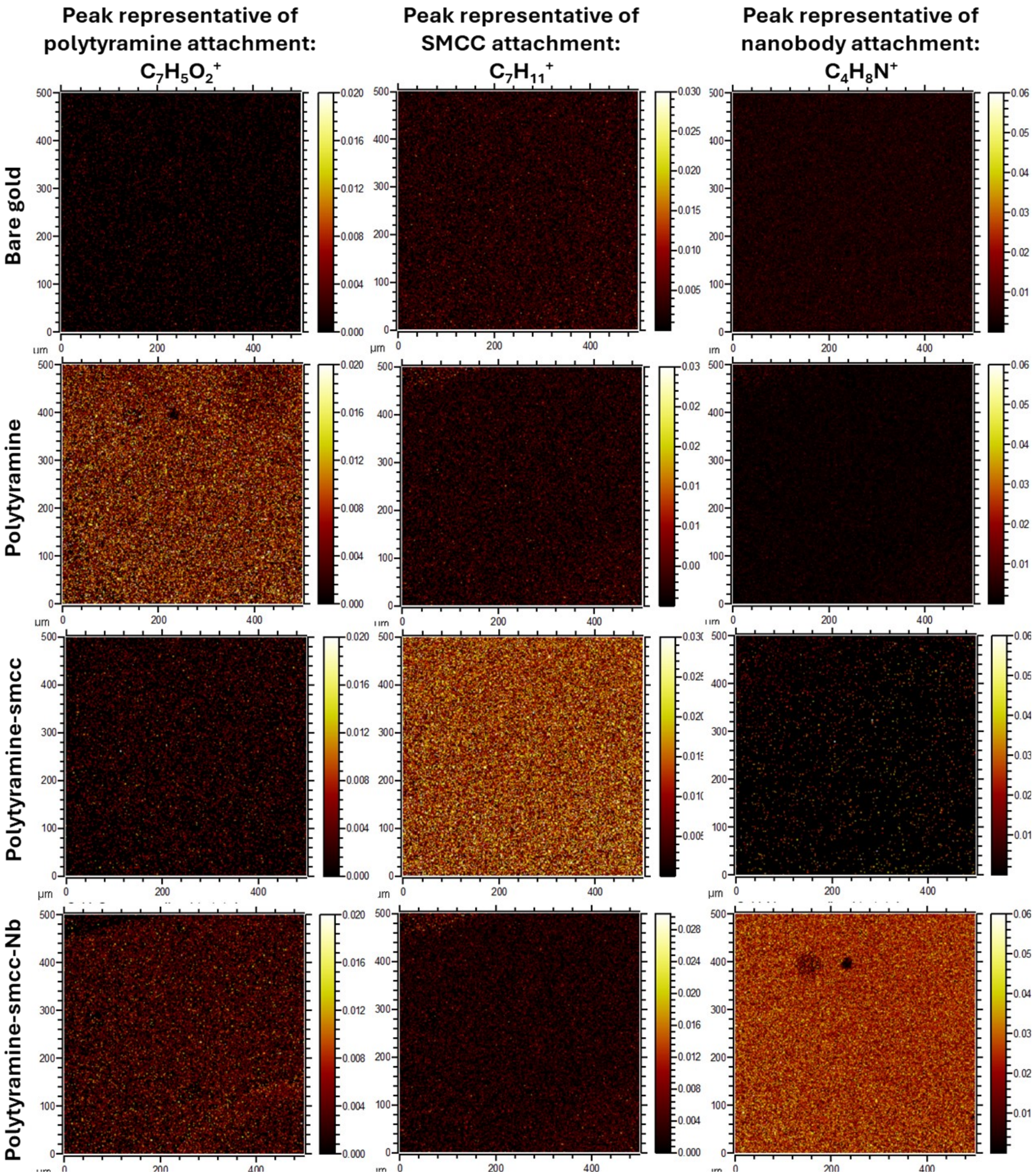

Figure S2 - Normalised ion images of the surface at each step of the functionalization. The chemistries characteristic to the functionalization have been identified using principal component analysis. Each column represents ion images representative of each functionalisation step and each row represents the sample type. Ion images are presented on the same intensity scale for each ion across the different samples.

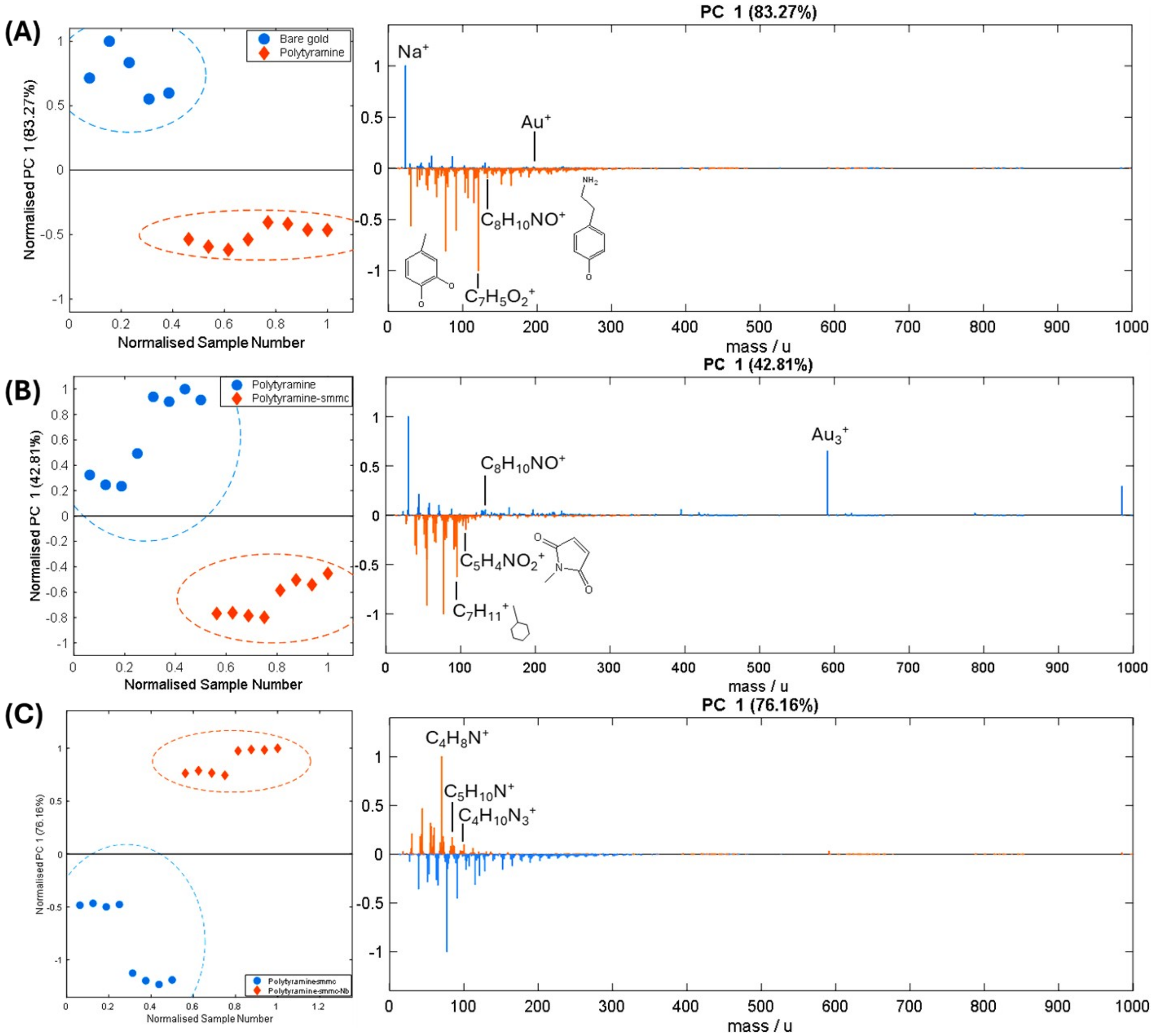

Figure S3 - Principal component analysis results at each step of the surface functionalization. **(A)** Bare gold and polytyramine functionalized surface, **(B)** polytyramine and polytyramine-SMCC functionalized surfaces and **(C)** polytyramine-SMCC-nanobody functionalized surface. At each step, the chemistries characteristic to the functionalization can be detected.

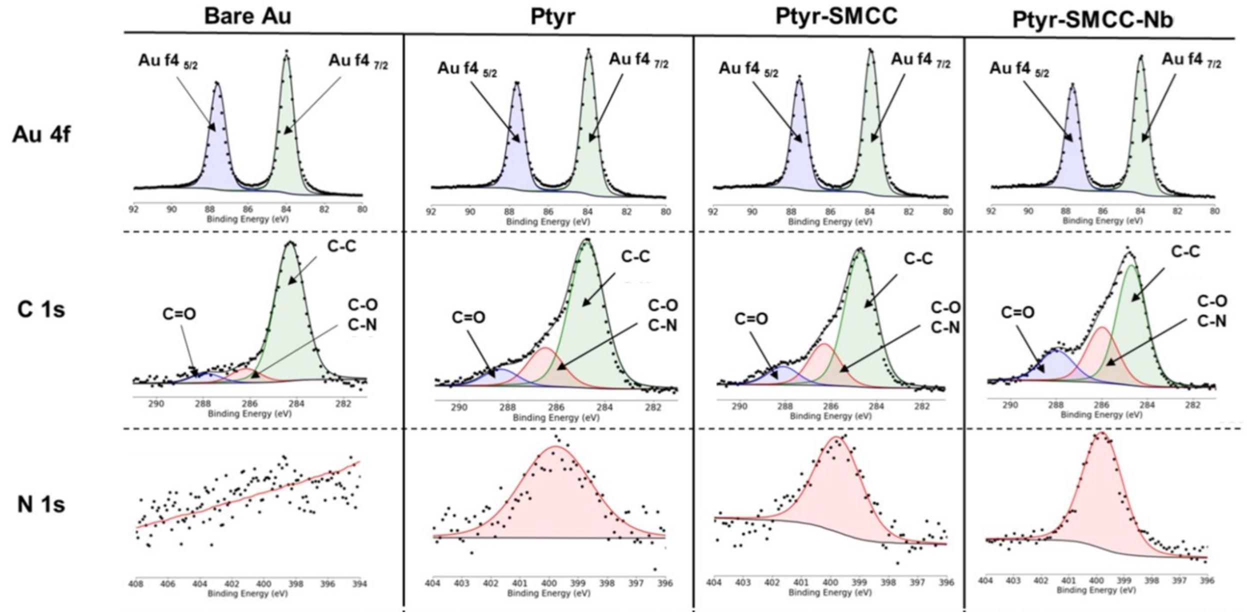

Figure S4 - High resolution spectra for the Au 4f, C 1s and N 1s XPS regions on bare Au, Ptyr, Ptyr-SMCC, and Ptyr-SMCC-Nb surfaces. Four measurements were carried out across two different samples.

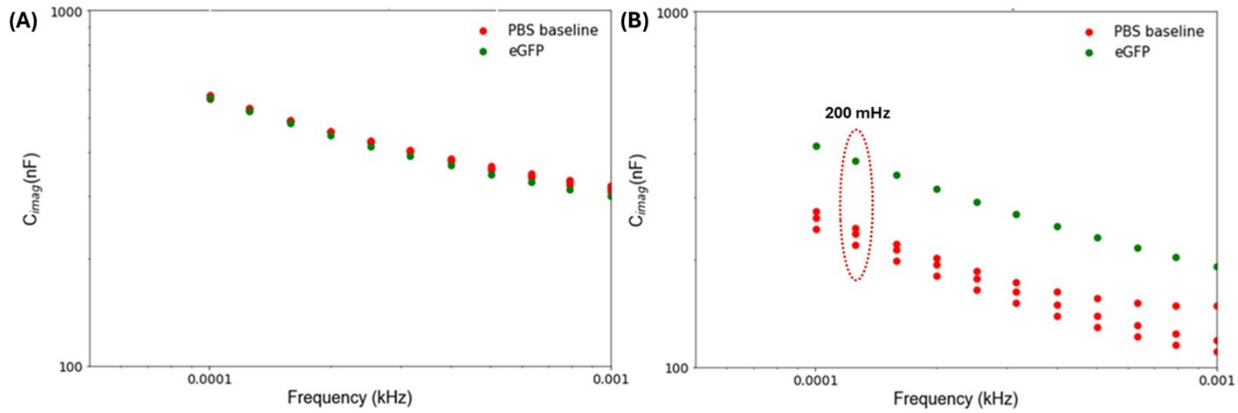

Figure S5 - Bode plots showing the low frequency range of the EIS spectrum when the measurement was carried out in **(A)** 1x PBS and **(B)** 0.001x PBS. Working electrode area = 0.03 cm^2.

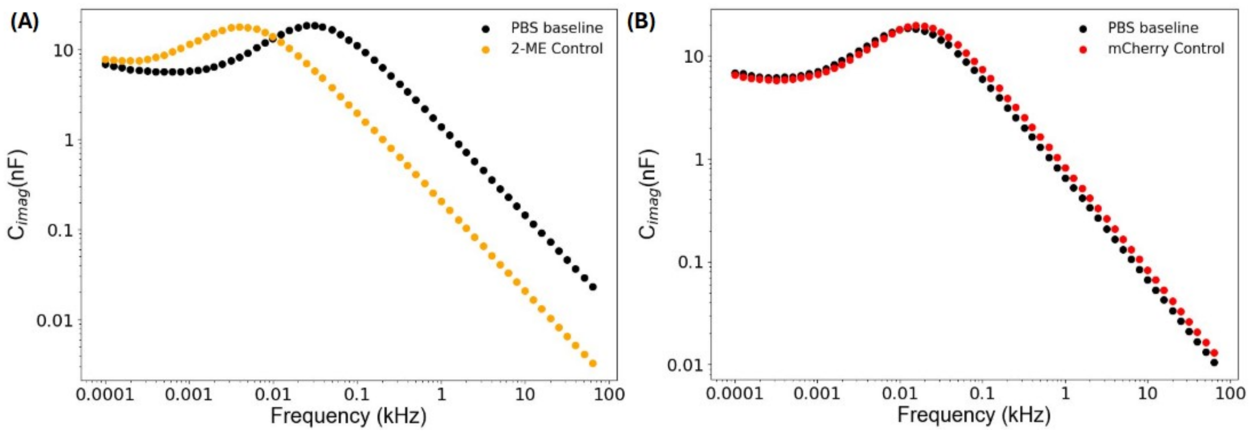

Figure S6 - (A) Bode plots for 2-mercaptoethanol (2-ME)-blocked surfaces before (black) and after eGFP incubation (yellow). **(B)** Bode plots before (black) and after non-target protein mCherry incubation (red). Working electrode area = 0.03 cm^2. The 2-ME blocking step passivates residual reactive surface sites and is not intended to form a thick dielectric layer. Therefore, only minimal changes in baseline impedance or capacitance are expected. Blocking effectiveness is reflected in the suppression of non-specific adsorption in subsequent measurements rather than in the magnitude of the blocking response.
